# Gut-kidney axis modulation by viable and inactivated *Akkermansia muciniphila* mitigates avian hyperuricemia through microbial-metabolic crosstalk

**DOI:** 10.1128/msystems.00773-25

**Published:** 2025-08-29

**Authors:** Yang Fu, Jiaqing Chen, Qingyun Cao, Shanshan Zhu, Wenjing Chen, Haotong Luo, Yue Zhao, Lukuyu A. Bernard, Xue Wang, Qiang Tu, Youming Zhang, Xianzhi Jiang, Ling Yang, Wence Wang

**Affiliations:** 1 State Key Laboratory of Swine and Poultry Breeding Industry, College of Animal Science, South China Agricultural University 12526, Guangzhou, China; 2 International Livestock Research Institute 54661, Nairobi, Kenya; 3 State Key Laboratory of Microbial Technology, Shandong University 520252, Qingdao, Shandong, China; 4 Shenzhen Key Laboratory of Genome Manipulation and Biosynthesis, CAS Key Laboratory of Quantitative Engineering Biology, Shenzhen Institute of Synthetic Biology, Shenzhen Institute of Advanced Technology, Chinese Academy of Sciences 85411 , Shenzhen, China; 5 Microbiome Research Center, Moon (Guangzhou) Biotech Co. Ltd., Guangzhou, China; 6 Gurdon Institute, University of Cambridge 2152, Cambridge, United Kingdom; APC Microbiome Ireland, Cork, Ireland

**Keywords:** hyperuricemia, uric acid metabolism, *Akkermansia muciniphila*, intestinal microbiota

## Abstract

Hyperuricemia (HUA) has become the fourth most important health-threatening risk factor after hypertension, hyperglycemia, and hyperlipidemia, but the efficacy of existing uric acid-lowering treatments (ULT) is poor, and there is an urgent need to explore novel ULT strategies. *Akkermansia muciniphila* (*A. muciniphila*), a next-generation probiotic, shows promise in promoting intestinal homeostasis and metabolic regulation. Previous studies have demonstrated the potential application of *A. muciniphila* in ULT, but its specific mechanism has not been elucidated. In this study, we isolated a strain of *A. muciniphila*, named K101, from the cecum of goslings. *In vitro* experiments showed that K101 directly degrades uric acid, suggesting a potential microbial-metabolic crosstalk mechanism for anti-HUA. *In vivo* experiments showed that K101 increased the abundance of uric acid metabolism-related microbiota, such as *A. muciniphila* and *Lactobacillus*. Functionally, K101 synergistically promoted uric acid excretion by activating the intestinal excretory protein ABCG2 and inhibiting the renal uric acid reabsorption protein GLUT9. In addition, K101 provides a stable environment for uric acid metabolism by inhibiting renal inflammatory responses. Overall, *A. muciniphila* K101 exerts anti-HUA effects by remodeling the intestinal microbiota and excretion of uric acid through the gut-renal axis. This study offers new insights into microbial-metabolic crosstalk in uric acid metabolism in *A. muciniphila* and identifies potential targets for gout prevention and ULT strategy development.

**IMPORTANCE:**

The rising prevalence of hyperuricemia (HUA) underscores the need for new therapies and treatment approaches. Our study highlights the developmental and therapeutic potential of natural uric acid-degrading bacteria discovered in the avian gut, expanding the range of bacteria with possible medical applications. Another key finding is the notable efficacy of microbiota metabolites in alleviating HUA. While the underlying mechanisms warrant further investigation, these findings offer promising insights into microbiota-based therapeutics.

## INTRODUCTION

Gout is a metabolic disorder resulting from abnormal purine metabolism. Hyperuricemia (HUA), characterized by elevated serum uric acid levels, is the biochemical cause of gout, leading to urate deposition in tissues and subsequent gout development (1). HUA is now a major health risk factor, ranking fourth after hypertension, hyperglycemia, and hyperlipidemia (2, 3). Gout, exacerbated by HUA, leads to high morbidity and mortality, particularly in goslings within poultry farming (4). Unlike most mammals, humans and birds lack uricase, making them prone to diet-induced HUA and gout (5). Geese, susceptible to HUA from high-protein diets and sharing similar purine metabolism and clinical signs with humans, present a more relevant and translatable model than murine models that rely on artificial induction and exhibit metabolic differences (4, 6). Consequently, building upon mechanism investigation and clinical analysis, we have developed an innovative and systematic assessment model for HUA to evaluate the uric acid-lowering effects of specific pharmaceuticals (4). Given the lack of effective treatments for HUA, there is a growing interest in exploring alternative therapeutic strategies, such as probiotics.

Probiotics are beneficial microorganisms that maintain intestinal microecological balance, enhance gut health, and contribute to immune regulation, metabolic balance, and nutrient absorption (7). In recent years, probiotics have gained attention as natural, non-toxic adjuvants for managing metabolic disorders, including HUA (8). Among these, *Akkermansia muciniphila* has emerged as a promising candidate due to its potential role in metabolic regulation.

*A. muciniphila* is a mucus-degrading bacterium that colonizes the mucin-rich mucus layer of the gut by utilizing mucin as its sole carbon and nitrogen source (9). Its abundance fluctuates with human host health and has been linked to metabolic diseases such as obesity, diabetes, and hypertension (10–12). In mouse models, *A. muciniphila* supplementation has been shown to improve metabolic disorders, intestinal inflammation, and neurodegenerative diseases (13, 14).

HUA is characterized by dynamic fluctuations in uric acid levels and intestinal barrier dysfunction, often accompanied by the depletion of beneficial bacteria such as *A. muciniphila* (15). Studies have demonstrated that interventions like inulin supplementation can enhance gut microbial diversity and increase *A. muciniphila* abundance, effectively alleviating HUA in urate oxidase (Uox) knockout mice (16). Additionally, *A. muciniphila* has been shown to mitigate HUA and improve gut microbiota composition in a mouse model (17). Our previous study also revealed that *A. muciniphila* was more abundant in the intestines of hyperuricemic goslings, suggesting its potential role in mitigating this condition (18). However, the precise mechanisms by which *A. muciniphila* regulates uric acid metabolism remain to be fully elucidated. Further investigation is warranted to determine whether *A. muciniphila* can contribute to reducing HUA in new models.

To further investigate its therapeutic potential, we isolated a strain of *A. muciniphila* from HUA goslings and designated it *A. muciniphila* K101. Our study revealed that *A. muciniphila* K101 effectively degraded uric acid *in vitro* during co-culture experiments. In a gosling model, live *A. muciniphila* K101, its pasteurized form, and metabolites all demonstrated significant efficacy in alleviating HUA. Mechanistically, *A. muciniphila* K101 promoted the expression of the intestinal uric acid excretion transporter ABCG2 while inhibiting the renal uric acid reabsorption protein GLUT9, collectively reducing blood uric acid levels. Additionally, *A. muciniphila* K101 modulated the host gut microbiota by increasing the abundance of beneficial bacteria, including *A. muciniphila*, *Lactobacillus*, and *Butyricicoccus*. Notably, the abundance of *A. muciniphila* exhibited a significant negative correlation with host blood uric acid levels. These findings elucidate the mechanisms by which *A. muciniphila* influences uric acid metabolism and provide a foundation for developing probiotic-based strategies to combat HUA. However, further safety assessments are needed for clinical trials.

## RESULTS

### *A. muciniphila* K101 derived from goose cecal chyme can degrade uric acid

**Fig 1:**
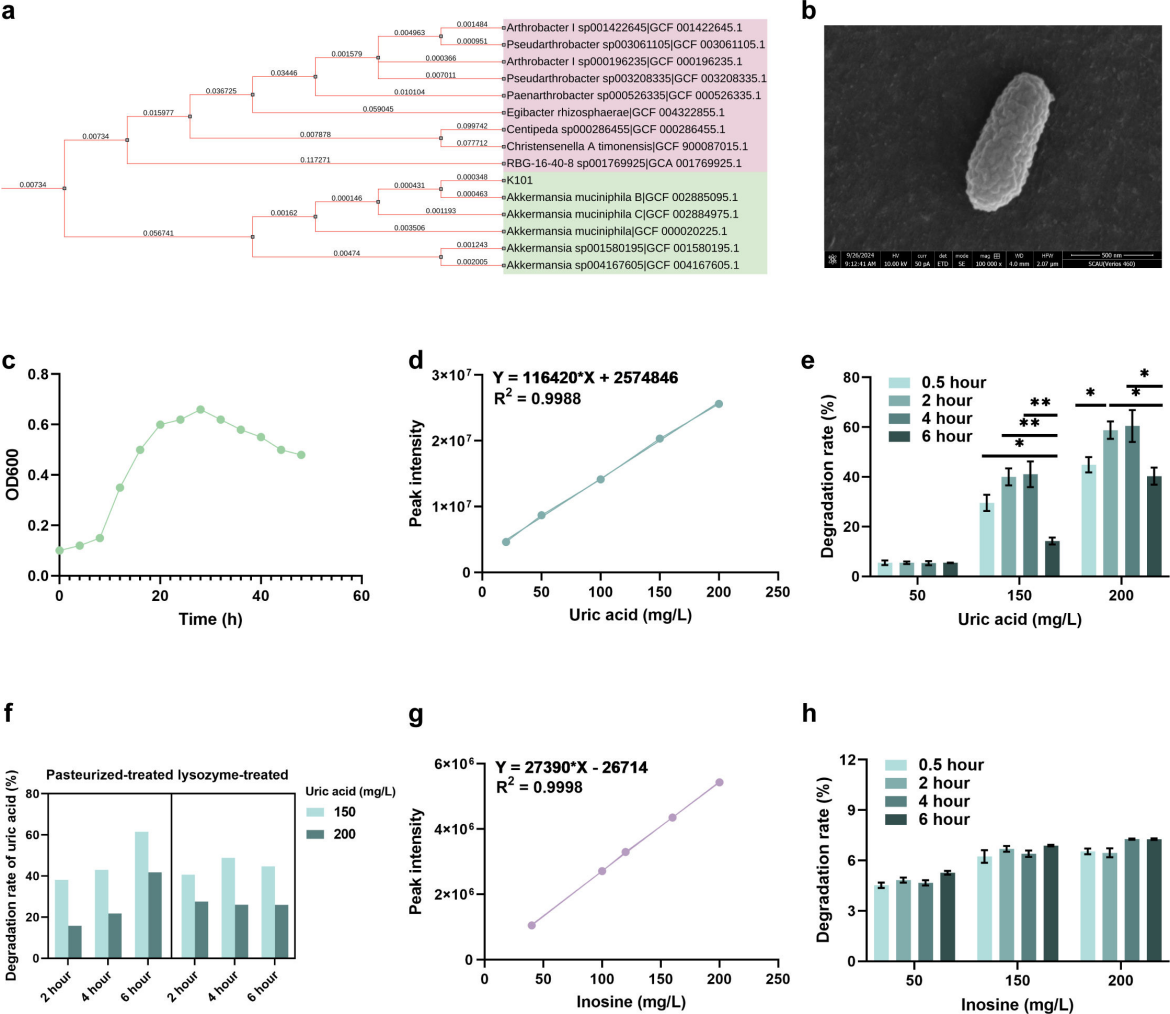
*Akkermansia muciniphila* K101 derived from goose cecal chow can degrade uric acid. (**a**) The phylogenetic tree of *A. muciniphila* K101. 16S rRNA of *A. muciniphila* K101 was extracted to sequence and blast the phylogeny, compared with 14 strains from different origins. Bootstrap values are displayed under the branches. (**b**) The transmission electron microscope results of the strain *A. muciniphila* K101. Magnification of 100,000. (**c**) Growth curve of *A. muciniphila* K101. (**d**) Standard curve of uric acid. (**e**) Degradation of uric acid by *A. muciniphila* K101 from HPLC analysis. (**f**) Degradation of uric acid by pasteurized-treated or lysozyme-treated *A. muciniphila* K101 from HPLC analysis. 150 and 200 represent uric acid level (mg/L). (**g**) Standard curve of inosine. (**h**) Degradation of inosine by *A. muciniphila* K101 from HPLC analysis. Statistical significance in panels **e and h** was determined by unpaired two-tailed Student’s *t*-test. *P < 0.05, **P < 0.01, and ***P < 0.001. Data with error bars represent mean ± S.E.M. K101, *A. muciniphila* K101.

We acquired a strain, *A. muciniphila* K101, from a cecal chyme sample collected from HUA geese. Comparing it with the 14 strains from the RefSeq, *A. muciniphila* K101 was closer to *A. muciniphila* B (GCF_002885095.1) (Fig. 1a). Query coverage reached 100%, with an alignment score of 2776. Scanning electron microscopy showed it to be long rod-shaped with a rough surface (Fig. 1b). To elucidate the mechanism by which *A. muciniphila* K101-mediated alleviation in HUA, the ability of *A. muciniphila* K101 to metabolize uric acid was initially investigated. The growth curve of *A. muciniphila* K101 was measured, and a standard curve for uric acid was constructed (Fig. 1c and d). Results indicated that live *A. muciniphila* K101 degraded 60% of uric acid in 4 h and 40% in 6 h (Fig. 1e). Pasteurized *A. muciniphila* K101 degraded 60% in 6 h, while lysozyme-treated *A. muciniphila* K101 degraded 40% in the same timeframe (Fig. 1f). The ability of *A. muciniphila* K101 to metabolize inosine was further tested. A standard curve for inosine was constructed (Fig. 1g). Results indicated that *A. muciniphila* K101 does not metabolize inosine, the precursor of uric acid production (Fig. 1h). These findings indicate that *A. muciniphila* K101 alleviates HUA by directly degrading uric acid rather than metabolizing its precursors.

### *A. muciniphila* K101 supplementation alleviates HUA in goslings

**Fig 2:**
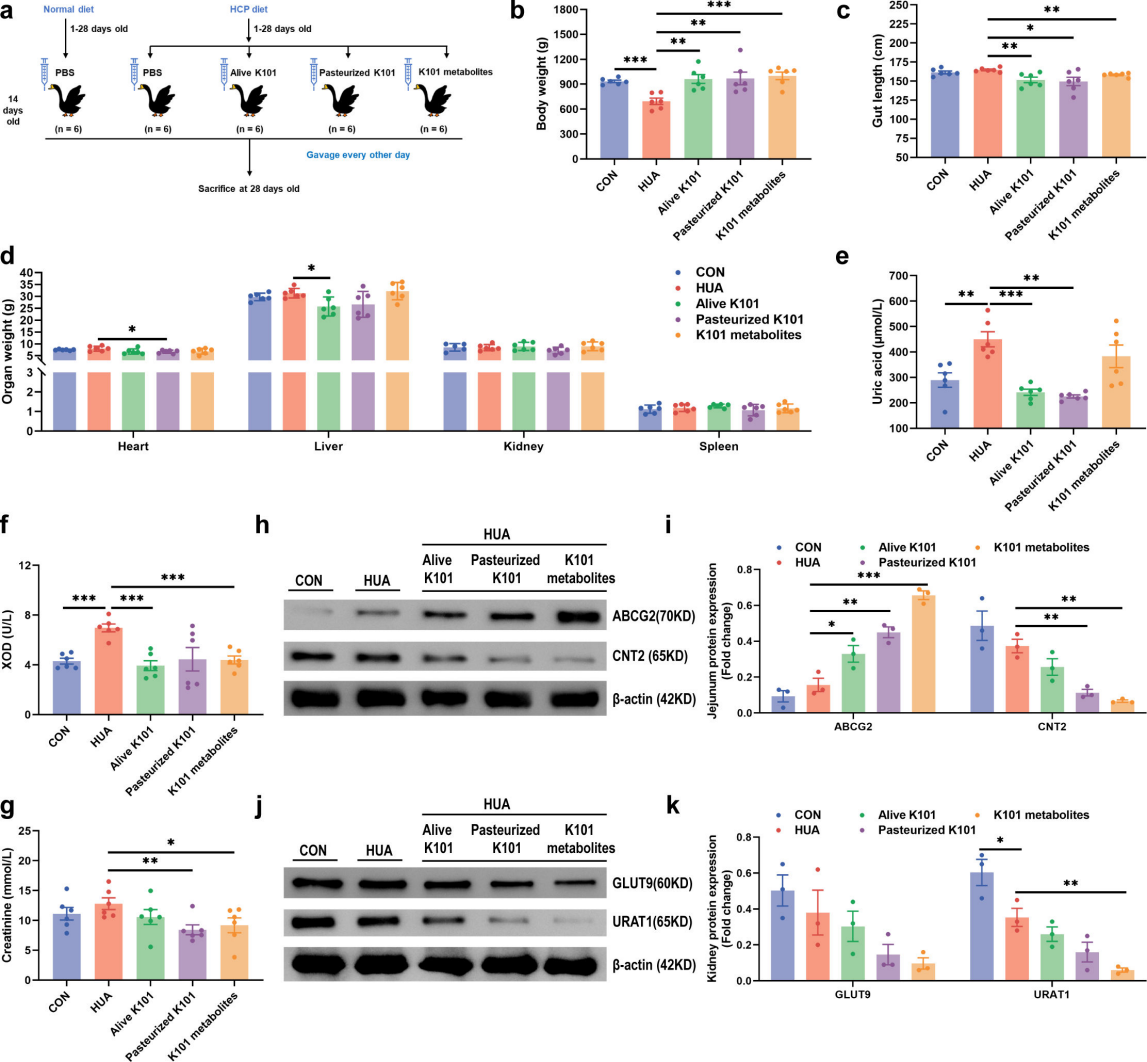
*Akkermansia muciniphila* K101 supplementation alleviates hyperuricemia (HUA) in goslings. (**a**) Experimental design. (**b**) Effect of Alive K101, Pasteurized K101*,* and K101 metabolites on the Final average weight in HUA geese (*n* = 6). (**c**) Effect of Alive K101, Pasteurized K101*,* and K101 metabolites on the gut length in HUA geese (*n* = 6). (**d**) Effect of Alive K101, Pasteurized K101*,* and K101 metabolites on the organ index in HUA geese (*n* = 6). (**e**) Effect of Alive K101, Pasteurized K101*,* and K101 metabolites on the serum uric acid in HUA geese (*n* = 6). (**f**) Effect of Alive K101, Pasteurized K101*,* and K101 metabolites on the serum XOD in HUA geese (*n* = 6). (**g**) Effect of Alive K101, Pasteurized K101*,* and K101 metabolites on the serum creatinine in HUA geese (*n* = 6). (**h**) Representative western blotting images and quantification of proteins (ABCG2, CNT2) in the jejunum tissue between the CON, HUA, Alive K101, Pasteurized K101*,* and K101 metabolites groups (*n* = 3). (**i**) Proteins (ABCG2, CNT2) expression level in the jejunum tissue between the CON, HUA, Alive K101, Pasteurized K101*,* and K101 metabolites groups (*n* = 3). (**j**) Representative western blotting images and quantification of proteins (GLUT9, URAT1) in the kidney tissue between the CON, HUA, Alive K101, Pasteurized K101, and K101 metabolites groups (*n* = 3). (k) Proteins (GLUT9, URAT1) expression level in the jejunum tissue between the CON, HUA, Alive K101, Pasteurized K101, and K101 metabolites groups (*n* = 3). Statistical significance in panels **b–g, i and k**) was determined by unpaired two-tailed Student’s *t*-test. *P < 0.05, **P < 0.01, and ***P < 0.001. Data with error bars represent mean ± S.E.M. K101, *A. muciniphila* K101. ABCG2, ATP-binding cassette transporter G2; CNT2, concentrative nucleoside transporter 2; GLUT9, glucose transporter 9; URAT1, urate transporter 1; XOD, xanthine oxidase.

To further confirm the functional role of *A. muciniphila* K101 in HUA, we administered live *A. muciniphila* K101 (Alive K101), pasteurized *A. muciniphila* K101 (Pasteurized K101), and *A. muciniphila* K101 metabolites (K101 metabolites) to HUA geese via oral gavage for 14 days, respectively (Fig. 2a). The results showed that Alive K101 (*P* < 0.01, *F* = 1.94, *U* = 0), Pasteurized K101 (*P* < 0.01, *F* = 4.03, *U* = 0), and K101 metabolites significantly improved final average weight (*P* < 0.001, *F* = 1.44, *U* = 0; Fig. 2b). Alive K101 (*P* < 0.01, *F* = 10.76, *U* = 1), Pasteurized K101 (*P* < 0.05, *F* = 25.37, *U* = 5), and K101 metabolites significantly decreased gut length (*P* < 0.01, *F* = 1.17, *U* = 0; Fig. 2c). Alive K101 decreased relative weight of liver (*P* < 0.05, *F* = 2.20, *U* = 0; Fig. 2d). Additionally, Alive K101 (*P* < 0.001, *F* = 5.69, *U* = 0), and Pasteurized K101 significantly reduced serum uric acid level (*P* < 0.01, *F* = 25.01, *U* = 0; Fig. 2e). Alive K101 (*P* < 0.001, *F* = 1.57, *U* = 0) and K101 metabolites (*P* < 0.001, *F* = 1.04, *U* = 0) significantly reduced xanthine oxidase (XOD) level (Fig. 2f). Pasteurized K101 (*P* < 0.01, *F* = 1.41, *U* = 0), and K101 metabolites (*P* < 0.05, *F* = 1.59, *U* = 0) significantly reduced creatinine contents (Fig. 2g).

Further analysis of key jejunum transporters involved in urate excretion revealed that Alive K101 (*P* < 0.05, *F* = 1.55, *U* = 0), Pasteurized K101 (*P* < 0.01, *F* = 1.47, *U* = 0), and K101 metabolites (*P* < 0.001, *F* = 2.38, *U* = 0) significantly increased the expression of ABCG2 (Fig.2h and i). Additionally, pasteurized K101 (*P* < 0.01, *F* = 4.07, *U* = 0), and K101 metabolites (*P* < 0.001, *F* = 31.75, *U* = 0) reduced the protein expression of concentrative nucleoside transporter 2 (CNT2) associated with uric acid formation in the jejunum (Fig. 2h and i). K101 metabolites reduced the protein expression of uric acid reabsorption in the kidney, including URAT1 (*P* < 0.01, *F* = 19.08, *U* = 0) (Fig. 2j and k). Collectively, *A. muciniphila* K101-mediated amelioration of HUA by decreasing uric acid production and enhancing uric acid excretion.

### *A. muciniphila* K101 supplementation reduced host inflammation

**Fig 3:**
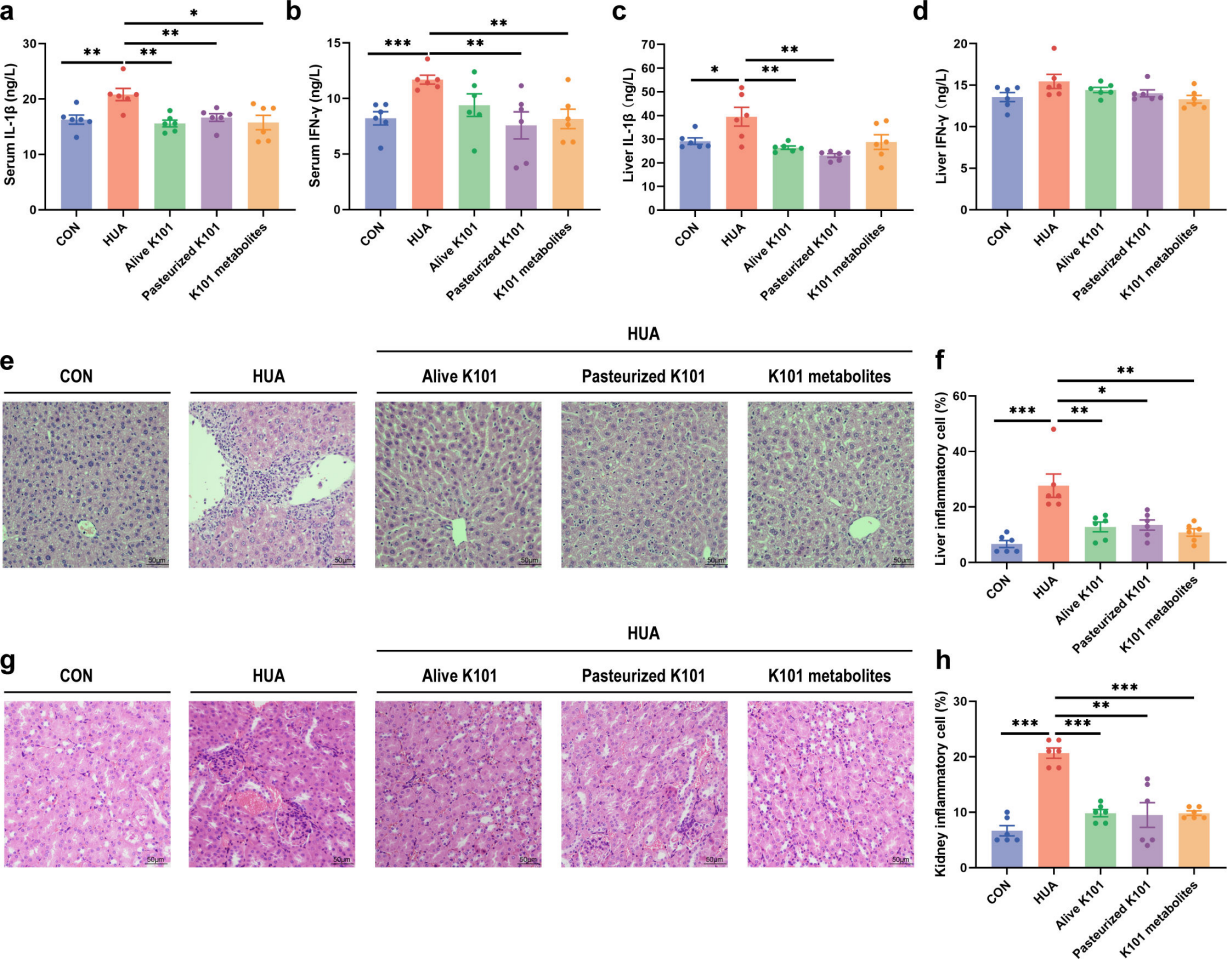
*Akkermansia muciniphila* K101 supplementation reduced host inflammation. (**a**) Effect of Alive K101, Pasteurized K101, and K101 metabolites on the serum IL-1β levels in HUA geese (*n* = 6). (**b**) Effect of Alive K101, Pasteurized K101, and K101 metabolites on the serum IFN-γ levels in HUA geese (*n* = 6). (**c**) Effect of Alive K101, Pasteurized K101, and K101 metabolites on the liver IL-1β levels in HUA geese (*n* = 6). (**d**) Effect of Alive K101, Pasteurized K101, and K101 metabolites on the liver IFN-γ levels in HUA geese (*n* = 6). (**e**) Representative H&E staining images and inflammatory cell rate of the liver section of geese (*n* = 6). Scale bar: 50 µm. (**f**) Inflammatory cell rate of the liver section of geese (*n* = 6). (**g**) Representative H&E staining images and inflammatory cell rate of kidney section of geese (*n* = 6). Scale bar: 50 µm. (**h**) Inflammatory cell rate of the kidney section of geese (*n* = 6). Statistical significance in panels **a–d, f, and h** was determined by unpaired two-tailed Student’s *t*-test. *P < 0.05, **P < 0.01, and ***P < 0.001. Data with error bars represent mean ± S.E.M. K101, *A. muciniphila* K101.

Additionally, Alive K101 (*P* < 0.01, *F* = 3.25, *U* = 0), Pasteurized K101 (*P* < 0.01, *F* = 2.46, *U* = 0), and K101 metabolites (*P* < 0.05, *F* = 1.41, *U* = 0) significantly decreased serum IL-1β level (Fig. 3a). Pasteurized K101 (*P* < 0.01, *F* = 9.26, *U* = 2) and K101 metabolites (*P* < 0.01, *F* = 4.71, *U* = 0) significantly decreased serum IFN-γ level (Fig. 3b). Alive K101 (*P* < 0.01, *F* = 28.89, *U* = 2), and Pasteurized K101 (*P* < 0.01, *F* = 25.34, *U* = 0) significantly decreased liver IL-1β levels (Fig. 3c). *A. muciniphila* K101 did not significantly affect liver IFN-γ expression (Fig. 3d). Furthermore, pathological sections indicated that live *A. muciniphila* K101, pasteurized *A. muciniphila* K101, and *A. muciniphila* K101 metabolites alleviated pathological damages like inflammatory cell infiltration in the liver (Fig. 3e and f) and kidney (Fig. 3g and h). These findings indicate that *A. muciniphila* K101 reduces inflammation in geese.

### *A. muciniphila* K101 supplementation modulates gut microbiome disturbances caused by HUA

**Fig 4:**
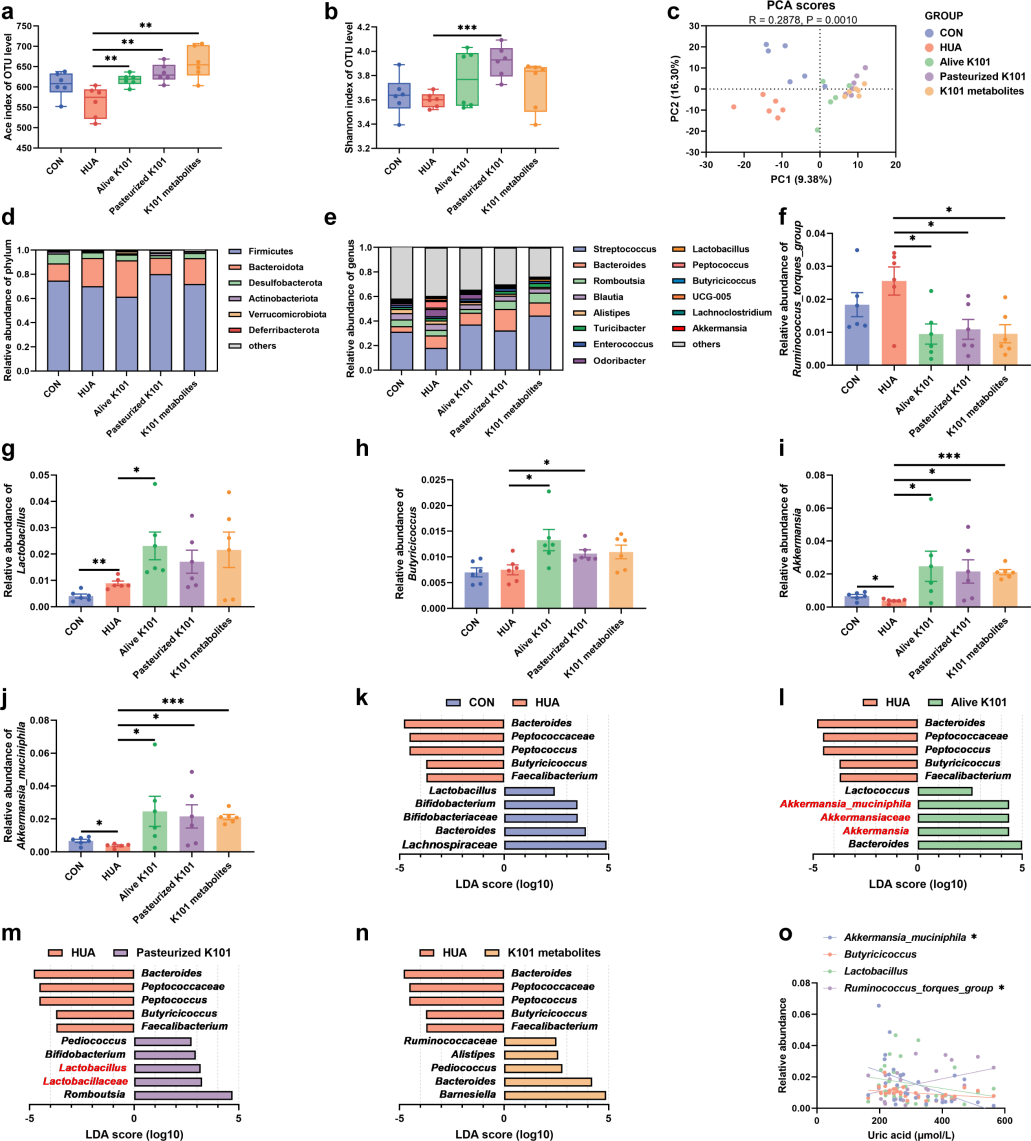
*Akkermansia muciniphila* K101 supplementation modulates gut microbiome disturbances caused by hyperuricemia (HUA). (**a**) Ace index of indicated groups based on alpha diversity analysis (*n* = 6). (**b**) Shannon index of indicated groups based on alpha diversity analysis (*n* = 6). (**c**) Principal component analysis of bacteria with 95% confidence regions of indicated groups (*n* = 6). (**d**) Relative abundance of gut microbes at the phylum levels of goose with indicated groups (*n* = 6). (**e**) Relative abundance of gut microbes at the genus levels of goose with indicated groups (*n* = 6). (**f**) Relative abundance of genus *Ruminococcus_torques_group* in HUA geese with indicated groups (*n* = 6). (**g**) Relative abundance of genus *Lactobacillus* in HUA geese with indicated groups (*n* = 6). (**h**) Relative abundance of genus *Butyricicoccus* in HUA geese with indicated groups (*n* = 6). (**i**) Relative abundance of genus *Akkermansia* in HUA geese with indicated groups (*n* = 6). (**j**) Relative abundance of species *A. muciniphila* in HUA geese with indicated groups (*n* = 6). (**k**) Linear discriminative analysis (LDA) score obtained from LEfSe of gut microbiota between HUA group and CON group (*n* = 6). Bacteria with Kruskal-Wallis ≤0.05, as well as LDA >2, are reported. (**l**) LDA score obtained from LEfSe of gut microbiota between Alive K101 group and HUA group (*n* = 6). Bacteria with Kruskal-Wallis ≤0.05, as well as LDA >2, are reported. (**m**) LDA score obtained from LEfSe of gut microbiota between Pasteurized K101 group and HUA group (*n* = 6). Bacteria with Kruskal-Wallis ≤0.05, as well as LDA >2, are reported. (**n**) LDA score obtained from LEfSe of gut microbiota between K101 metabolites group and HUA group (*n* = 6). Bacteria with Kruskal-Wallis ≤0.05, as well as LDA >2, are reported. (**o**) Spearman correlation analysis between serum uric acid and gut microbes. Statistical significance in panels **a, b, and f–j** was determined by unpaired two-tailed Student’s *t*-test. *P < 0.05, **P < 0.01, and ***P < 0.001. Data with error bars represent mean ± S.E.M. K101, *A. muciniphila* K101.

Sequencing analysis of cecal chyme revealed that both Alive K101 (*P* < 0.01, *F* = 7.04, *U* = 0), Pasteurized K101 (*P* < 0.01, *F* = 2.82, *U* = 0), and K101 metabolites (*P* < 0.01, *F* = 1.08, *U* = 0) significantly increased microbiota richness (Ace index) compared to the HUA group (Fig. 4a). Additionally, Pasteurized K101 significantly increased microbiota uniformity (Shannon index, *P* < 0.001, *F* = 4.74, *U* = 0) compared to the HUA group (Fig. 4b). Principal component analysis (PCA) showed the separation of gut microbiota in Alive K101, Pasteurized K101, K101 metabolites, and HUA groups (Fig. 4c; Fig. S1). Sequencing results showed that Alive K101 enhanced the relative abundance of phylum *Verrucomicrobia*, Pasteurized K101, and K101 metabolites enhanced the relative abundance of phylum *Fimicutes* (Fig. 4d). Meanwhile, the relative abundance of genus *Ruminococcus*_*torques*_*group*, *Lactobacillus*, *Butyricicoccus*, *Akkermansia*, and species *A. muciniphila* was also markedly boosted (Fig. 4e through j). The linear discriminant analysis (LDA) effect size (LEfSe) revealed that (LDA > 2), compared to the HUA group, the key differential bacteria in the Alive K101 group were the genus *Akkermansia* and species *A. muciniphila*, while in the Pasteurized K101 group, they were primarily *Lactobacillus*, and in the K101 metabolites group, the genus *Barnesiella* (Fig. 4k through n).

Additionally, Spearman correlation analysis revealed a significant negative association between *A. muciniphila* (*R* = 0.226, *Y* = −6.976e−005 *× X* + 0.03751, *P* < 0.05, *F* = 7.62) and serum uric acid levels, while the *Ruminococcus*_*torques*_*group* exhibited a strong positive correlation with uric acid (*R* = 0.175, *Y* = 4.073e−005 × *X* + 0.002485, *P* < 0.05, *F* = 5.71; Fig. 4h). Overall, *A. muciniphila* K101 ameliorated HUA-induced gut microbiological disturbances, restored and increased the abundance of *Lactobacillus* spp., *Butyricicoccus* spp., and *Akkermansia* spp., and ultimately alleviated HUA in geese.

## DISCUSSION

Research indicates that gut microbiota experiences dynamic changes during the development of HUA, with microbial dysbiosis worsening as the disease advances. In HUA model mice, fluctuations in uric acid levels correlated negatively with the depletion of the beneficial bacterium *A. muciniphila* (15). However, in another study, the abundance of *A. muciniphila* was increased in the intestines of HUA model mice (19, 20). Moreover, administration of a novel hexapeptide derived from *Apostichopus japonicus* hydrolysate further increased *A. muciniphila* abundance and alleviated HUA. Correlation analyzes also showed a significant negative correlation between *A. muciniphila* and uric acid levels (19). These studies suggest that *A. muciniphila* may play a key role in HUA development and progression, but its specific function and mechanism of action need to be further verified. Our previous study found that *A. muciniphila* abundance was increased in the intestines of HUA goslings, but the relationship between *A. muciniphila* and HUA is still unclear (18). Therefore, we isolated a strain of *A. muciniphila*, K101, from cecal chyme of HUA goslings and further investigated the specific function and mechanism of *A. muciniphila*.

Numerous studies indicate that beneficial bacteria, including *Lactobacillus plantarum*, *Lactobacillus rhamnosus*, *Lactobacillus fermentum*, and *Lactobacillus paracasei*, can lower uric acid levels (21–24). They primarily achieve this by degrading uric acid or its precursors, or by competing with the intestinal epithelium for uric acid uptake (23). Our study showed that co-culture with uric acid or inosine, the uric acid degradation rate of *A. muciniphila* could reach 60%, and with higher uric acid concentration, *A. muciniphila* degradation rate was higher. However, *A. muciniphila* failed to degrade inosine. In addition, pasteurized and lysozyme-treated *A. muciniphila* had the same degradation effect on uric acid. But the effect of pasteurized treatment was better than that of lysozyme treatment, which might be related to the fact that lysozyme destroyed the cellular structure of the bacteria.

In the *in vivo* test, *A. muciniphila*-treated geese showed a significant improvement in body weight compared to the HUA group. Body weight data were calculated as the average weight of each replicate, a standard method widely applied in livestock trials to ensure accuracy and reproducibility (25–28). The geese closest to the average weight in each replicate were selected for sampling in the follow-up trials (*n* = 6 per group).

The intestine and kidneys play a vital role in uric acid excretion. Research indicates that both live and pasteurized *A. muciniphila* reduce HUA in mice by modulating the expression of uric acid transporters ABCG2 and GLUT9 in the intestine and kidneys (17). This study found that both live and pasteurized K101 and K101 metabolites increased the intestinal uric acid excretory protein ABCG2 and decreased the renal uric acid reabsorption protein GLUT9, thereby reducing HUA in goslings. CNT2 is a key purine nucleoside transporter primarily responsible for their intestinal uptake and is highly expressed in the kidney, ileum, jejunum, and other organs, supplying raw materials for uric acid synthesis in the liver (18, 29). The present findings suggest that oral administration of live and pasteurized K101, and K101 metabolites downregulated intestinal CNT2 expression, further preventing the accumulation of uric acid.

The study found that serum and liver levels of inflammatory cytokines (IL-1β and INF-γ) were significantly elevated in the HUA group compared to the CON group, indicating that hyperuricemic goslings experienced low-grade systemic inflammation. Inflammatory cytokines have been reported to upregulate the expression and activity of hepatic XOD, which is a key enzyme that promotes uric acid production (16, 30, 31). The present findings suggested that XOD activity was elevated in the HUA group, whereas oral administration of live and pasteurized K101 and K101 metabolites reduced XOD levels.

Several studies have demonstrated that gut microbiota represent an important link to HUA. Bacterial diversity and structure are altered during the progression of HUA, and the dysbiosis worsens further with HUA (15, 32, 33). This study noted that *A. muciniphila* abundance was lowered in the intestine of HUA goslings and increased by oral administration of live and pasteurized K101 and K101 metabolites. Meanwhile, the abundance of the beneficial bacteria *Lactobacillus* and *Bifidobacterium* was also increased. LEFSe revealed that these beneficial bacteria were predominant in both the live and pasteurized K101 groups, significantly alleviating host HUA. In our previous study, we found a significant increase in *Ruminococcus_torques_group* in the HUA group, and this was also observed in the present study (4, 18). Its functions appear varied, as *Ruminococcus_torques_group* is associated with butyrate production and plays a key role in degrading intestinal mucin glycoproteins (34–36). However, *Ruminococcus_torques_group* has also been associated with the development of intestinal diseases, showing significantly higher abundance in patients with Crohn’s disease (CD) and inflammatory bowel disease (IBD) (37, 38). Our results indicate that *Ruminococcus_torques_group* supports the development of HUA, while the oral administration of live and pasteurized K101, as well as its metabolites, decreased its abundance. Spearman’s correlation analysis revealed a significant positive correlation between *Ruminococcus_torques_group* and uric acid, whereas *A. muciniphila* showed a significant negative correlation with uric acid.

### Conclusion

In this study, we isolated a novel strain of *A. muciniphila* K101 and used it as a functional additive to study its effects on gut microbiota composition and uric acid metabolism in hyperuricemic goslings. Oral administration of *A. muciniphila* K101 led to a more balanced and beneficial gut microbiota by inhibiting *Ruminococcus_torques_group* and promoting the colonization of *A. muciniphila*, *Lactobacillus*, and *Butyricicoccus*. The modified microbiota enhanced intestinal uric acid metabolism through microbiome-host crosstalk. Simultaneously, *A. muciniphila* K101 modulated host health by reducing inflammatory factors. These changes ultimately alleviated HUA in goslings. In conclusion, this study enhances our understanding of gut microbiota’s significant role in uric acid metabolism, thereby supporting the use of *A. muciniphila* K101 in gosling production. Avian gut bacteria capable of degrading uric acid show developmental and therapeutic promise, broadening the scope of bacteria with potential medical uses. However, further safety assessments are needed for clinical trials.

## MATERIALS AND METHODS

### Animal management

The present study replicated established protocols from prior research (3). Male goslings (post-hatch day 1) were sourced from a certified breeding facility in Qingyuan, Guangdong Province, China. Experimental cohorts were group-housed in five-bird units within stainless steel enclosures with ad libitum provision of feed and drinking water. Thermal regulation parameters followed a phased protocol: initial heating cycle at 33 ± 1°C subjected to progressive weekly decrements of 2.5 ± 0.5°C until achieving thermal equilibrium at 26°C. Ambient humidity levels were maintained within 45–60% range throughout the trial period.

### Live *A. muciniphila* K101, pasteurized *A. muciniphila* K101, and *A*. *muciniphila* K101 metabolites supplementation in HUA goslings

The experimental protocol followed the methodology previously outlined (3). A total of 150 one-day-old male geese were divided into two initial groups: the control (CON, Group A) and the HUA (Group B) groups. After 14 days of HUA modeling, Group B was further subdivided into four subgroups (B, C, D, and E). From days 14 to 28, Groups A and B received phosphate-buffered saline (PBS), while Groups C, D, and E were administered live *A. muciniphila* K101 bacteria, pasteurized *A. muciniphila* K101 bacteria, and *A. muciniphila* K101 supernatant metabolites, respectively, all resuspended in PBS at a concentration >1 × 10^9^ CFU. Each group has six replicates and five geese for each replicate, with one animal selected from each replicate based on the closest average body weight. *A. muciniphila* was cultured for 24 h, and its optical density (OD) and colony-forming units (CFU) were measured. The CFU was adjusted to 1 × 10^9^ CFU/mL. Following centrifugation at 5,000 × *g*, the supernatant was collected as *A. muciniphila* metabolite, and the pellet was resuspended in PBS as live *A. muciniphila*. The resuspended *A. muciniphila* was then pasteurized by heating to 70°C for 30 min as pasteurized *A. muciniphila*. At 28 days, one goose closest to average body weight from each replicate across all groups was euthanized for analysis.

### Sample collection

The experimental procedure followed the same methodology as previously described (3). Blood samples were obtained from each goose, and serum isolation was achieved through centrifugation (1,200 × *g*, 10 min, 4°C) prior to −30°C cryopreservation for biochemical profiling. Complete visceral dissection, including heart, liver, kidney, and spleen, which were collected and weighed. Intestinal tract dimensions were recorded. Liver and kidney (approximately 1–2 cm³) were obtained after rinsing with PBS. Cecum chyme was snap-frozen at −80°C. Animal body weights were surveillance tracked throughout the intervention.

### Analysis of biochemical parameters

The experimental protocol followed the methodology previously outlined (3). Serum levels of uric acid, XOD, and creatinine were quantified using biochemical kits (Jiancheng Biotechnology, Nanjing, China). Additionally, interleukin-1β (IL-1β) and interferon-γ (IFN-γ) levels were measured using an Enzyme-Linked Immunosorbent Assay (ELISA) Kit (Meimian Industrial, Jiangsu, China) following standardized protocols.

### Western blot analysis

Immunoblotting analyzes were conducted following established procedures (3). Tissue homogenates from jejunal and hepatic specimens were digested in RIPA buffer (Sigma-Aldrich, St. Louis, MO, USA). Protein quantification was performed using a bicinchoninic acid assay system (Thermo Fisher Scientific). Denaturation was achieved by thermal processing at 100°C for 600 s after mixing lysates with 5× loading buffer (4:1 [vol/vol]). Electrophoretic separation was performed on 10% SDS-PAGE gels, followed by electrotransfer to polyvinylidene fluoride matrices. Membranes underwent sequential incubation cycles: primary antibody treatment (16–18 h, 4°C) and species-matched immunoglobulin G conjugated with horseradish peroxidase (1:5,000, 2 h, 25°C). Chemiluminescent signals were captured using a Bio-Rad imaging platform and quantified via ImageJ software with β-actin for OD normalization. Primary antibodies included β-actin (1:5,000, 66009-1-Ig, Proteintech, USA), ABCG2 (1:1,000, 10051-1-AP, Proteintech, USA), CNT2 (1:500, DF4522, Affinity Biosciences, USA), GLUT9 (1:500, 26486-1-AP, Proteintech, USA), and URAT1 (1:500, 14937-1-AP, Proteintech, USA). Secondary antibodies were anti-mouse IgG-HRP (AWS0001, 1:5,000, Abiowell, China) and anti-rabbit IgG-HRP (AWS0002, 1:5,000, Abiowell, China). β-Actin served as the loading control for protein normalization.

### 16S rRNA sequencing

Cecal chyme microbiological investigations were conducted following the referenced protocol (3). Purified PCR amplicons targeting the V3–V4 hypervariable regions of the 16S rRNA gene were pooled equimolarly and sequenced using Illumina MiSeq PE300 or NovaSeq PE250 platforms (Illumina, San Diego, USA) by Majorbio Bio-Pharm Technology Co., Ltd. (Shanghai, China). Raw sequencing reads were demultiplexed and quality-filtered using fastp (v0.20.0), followed by paired-end read merging with FLASH (v1.2.7) under strict criteria: truncation of reads with a 50 bp sliding window average quality score < 20, removal of sequences <50 bp or containing ambiguous bases, assembly of only those with >10 bp overlap and mismatch ratio < 0.2, and discarding unassembled reads. Barcode and primer processing required exact barcode matching and allowed ≤2 primer mismatches. Operational taxonomic units were clustered at 97% sequence similarity using UPARSE (v7.1), with chimeric sequences removed. Taxonomic annotation was performed using the RDP Classifier (v2.2) against the SILVA 16S rRNA database (confidence threshold 0.7). For microbial community analysis, diversity indices (Shannon/Ace) were computed to characterize alpha diversity, while intergroup structural variation was assessed using PCA based on the Euclidean distance algorithm, combined with a test method (Anosim) to assess whether this community structure change was significant (*P* < 0.05). Differential species were identified using rank-sum tests with FDR correction, and bacterial biomarkers were determined using LEfSe with an LDA score > 2. Metabolic interaction networks between gut microbiota and serum metabolites were constructed using Spearman’s correlation analysis. The data were analyzed on the online platform of Majorbio Cloud Platform (https://www.majorbio.com/).

### Strains and media

Experimental protocols were established per cited methodology (3). The *A. muciniphila* K101 strain (accession: CGMCC 40993) was sourced from the Waterfowl Nutrition Research Center (South China Agricultural University, Guangzhou, China). Brain-heart infusion (BHI) broth (HB8442, Huankai Microbial, Guangdong) and porcine mucin (Catalog no. M2378; Sigma-Aldrich) served as culture substrates. Cecal digesta underwent serial 10-fold dilution (10^−1^ to 10^−9^) within an aseptic laminar flow hood. Discrete colonies were subcultured in nutrient broth for genomic DNA isolation. Genomic DNA was extracted using a DNA extraction kit (magnetic beads) (Majorbio, Shanghai, China), followed by amplification with universal primers, and then sent to Sangon Biotech (Shanghai, China) for sequencing. After sequencing, bacterial strains were identified by aligning the sequences with the GenBank database using BLASTn. Based on the alignment results, 15 strains of bacteria that were closest to each other at the species level were selected, and the neighbor-joining (NJ) method was used to construct the phylogenetic tree. Seed cultures were prepared in mucin-supplemented BHI medium (3% [wt/vol]) through iterative passaging: Initial propagation at 5% inoculum for three successive growth cycles, subsequently scaled to 10% inoculum for experimental applications. All cultures were maintained at 37°C under microaerophilic conditions with 18-h incubation intervals.

### Determination of uric acid degradation rate

Modified protocols from Fu et al. were implemented (3). Urate working solution was formulated by dissolving 20 mg urate crystals in 100 mL phosphate buffer (10 mM K_3_PO_4_, pH 7.0). Calibration standards (0.025–0.2 g/L) were prepared through the serial dilution method, with analyte quantification performed using external standard calibration on the HPLC system (Waters e2695). Twenty-four-hour bacterial cultures (10 mL) were subjected to refrigerated centrifugation (6,000 × *g*, 10 min, 4°C; *n* = 3), followed by sequential PBS rinses. The resulting cell pellets were reconstituted in 750 μL urate solution for oxygen-limited incubation (37°C, 120 rpm orbital shaker). Kinetic sampling was conducted at pre-defined intervals (0.5–6 h) with triplicate biological replicates per time point.

### Determination of nucleoside degradation rate

Experimental procedures were modified from established protocols (3). An aqueous nucleoside formulation was prepared by dissolving 20 mg inosine (Sigma-Aldrich I4125) in potassium phosphate buffer (10 mM, pH 7.0) to achieve 0.2 g/L working concentration. Five-point calibration standards (0.04–0.2 g/L) were established using linear regression analysis (*R*² > 0.998) through external standard HPLC quantification. Stational-phase cultures (*n* = 3 independent replicates) underwent refrigerated centrifugation (6,000 × *g*, 10 min, 4°C) with subsequent PBS washing cycles (1 mL × 2). Processed microbial pellets were suspended in 750 μL nucleoside solution under oxygen-limited incubation conditions (37°C, 120 rpm). Kinetic sampling was performed at designated intervals (1/3/6 h) with biological triplicates maintained for each time point.

### HPLC determination

Experimental workflow was executed according to established parameters from the reference method (3). Processed samples underwent refrigerated centrifugation (4,000 × *g*, 10 min, 4°C), followed by precise aliquot mixing: 720 μL clarified supernatant combined with 80 μL ice-cold 0.1 M HClO_4_ (prepared in deionized water) maintaining 9:1 acidification ratio. Membrane-filtered (0.22 μm pore size, sterile PVDF membrane) extracts were analyzed by HPLC-UV (Waters e2695) using Spursil EP C18 column (250 × 4.6 mm, 5 μm particle size). Isocratic elution employed mobile phase containing methanol-modified phosphate buffer (20 mM KH_2_PO_4_, pH 3.0; methanol 1% vol/vol) with 1.0 mL/min flow rate. Chromatographic separation occurred in isothermal column compartment (25°C) coupled with UV-Vis detection at *λ* = 254 nm, using elution gradient spanning 30 min.

### Statistical analysis

All quantitative results are expressed as arithmetic mean ± standard error unless specifically annotated. Statistical operations were performed using SPSS Statistics (Version 26.0, IBM Corporation). Parametric testing (unpaired *t*-test) or nonparametric analysis (Mann-Whitney *U*-test) was applied for dual-group comparisons after verifying data distribution normality. Statistical significance thresholds were defined as follows: *P* < 0.05; *, *P* < 0.05; **, *P* < 0.01; ***, *P* < 0.001; and ****, *P* < 0.0001.

## Data Availability

The 16S rRNA sequencing data for all samples have been deposited into the NCBI Sequence Read Archive database (project number PRJNA1193115).

## SUPPLEMENTAL MATERIAL

Supplemental material msystems.00773-25-s0001.xlsx Data for Fig. 1 to 4; Fig. S1.

ASM does not own the copyrights to Supplemental Material that may be linked to, or accessed through, an article. The authors have granted ASM a non-exclusive, world-wide license to publish the Supplemental Material files. Please contact the corresponding author directly for reuse.
